# Supplementary material for: Marine n-3 fatty acid consumption in a Norwegian renal transplant cohort: Comparison of a food frequency questionnaire with plasma phospholipid marine n-3 levels
Source: PLoS One. 2020 Dec 17;15(12):e0244089. doi: 10.1371/journal.pone.0244089 (PMC7746258; doi:10.1371/journal.pone.0244089)
Supplement: S1 Fig — The study subjects responded to the question “During a typical month, how often do you eat these food items?” using one of six response alternatives for each of the ten food item categories. (PDF) [file pone.0244089.s001.pdf]

KOSTHOLDSREGISTRERING | **Randomiseringsnummer** ☐☐☐

ORENTRA studien | **Pasient initialer** ☐☐☐

Dato (dag/måned/år):

				2	0	1	

I forbindelse med deltakelse i studien bes du svare på disse 10 spørsmålene som kartlegger dine kostholdsvaner mhp inntak av omega-3 fettsyrer. Skjemaet fylles ut sammen med utprøver og det skal kun settes ett kryss pr. spørsmål. Dersom du er usikker på hva du vanligvis spiser i løpet av en måned, ta utgangspunkt i siste måned. Svar så ærlig som mulig. Deltakelse er frivillig.

	I løpet av en vanlig måned, hvor ofte spiser du disse matvarene?	Aldri	Sjelden	1-2 ganger pr. mnd	3-4 ganger pr. mnd	2-3 ganger pr. uke	Mer enn 3 ganger pr. uke
1	Sild til middag?	☐	☐	☐	☐	☐	☐
2	Fete fiskeslag som laks, ørret, sardiner eller makrell til middag?	☐	☐	☐	☐	☐	☐
3	Tunfisk eller hellefisk som kveite, rødspette og flyndre til middag?	☐	☐	☐	☐	☐	☐
4	Magre fiskeslag som uer, steinbit, lyr, torsk og sei til middag?	☐	☐	☐	☐	☐	☐
5	Mat laget av fiskefarse, fiskegrateng og panert fisk til middag?	☐	☐	☐	☐	☐	☐
6	Annen sjømat som krabbe, blåskjell, reker og hummer til middag?	☐	☐	☐	☐	☐	☐
7	Fet fisk (sild, laks, makrell, sardiner og anjos) som pålegg?	☐	☐	☐	☐	☐	☐
8	Mager fisk (torsk, tunfisk og mat laget av fiskefarse) som pålegg?	☐	☐	☐	☐	☐	☐
9	Annen sjømat (krabbe, reker og krepsehaler) som pålegg?	☐	☐	☐	☐	☐	☐
10	Tran eller omega-3 i flytende form eller som kapsler?	☐	☐	☐	☐	☐	☐

Takk for at du tok deg tid til å svare på spørsmålene!

Med vennlig hilsen
Ivar Anders Eide på vegne av prosjektgruppen
