# Supplementary material for: Marine n-3 fatty acid consumption in a Norwegian renal transplant cohort: Comparison of a food frequency questionnaire with plasma phospholipid marine n-3 levels
Source: PLoS One. 2020 Dec 17;15(12):e0244089. doi: 10.1371/journal.pone.0244089 (PMC7746258; doi:10.1371/journal.pone.0244089)
Supplement: S2 Fig — The study subjects responded to the question “During a typical month, how often do you eat these food items?” using one of six response alternatives for each of the ten food item categories. Based on EPA and DHA content in the meat of various fish and other seafoods presented in the US Department of Agriculture Food Composition Database and assuming a standard portion size for dinner and bread spread, every potential response was given a weight (shown inside boxes). Total intake of marine n-3 fatty acids per month was calculated as the sum of the ten weighted responses in grams. (PDF) [file pone.0244089.s002.pdf]

Food frequency questionnaire for the ORENTRA study

Randomization number	
Patient initials	
Date (day/month/year)	_ _ / _ _ / 2 0 _ _

In conjunction with your participation in the ORENTRA study, we ask you to answer the 10 questions below about your eating habits in regards to omega-3 fatty acid intake. Please choose only one answer per question. If you are uncertain about what you eat in a typical month, base your answers on your food intake in the past month. Please answer as honestly as possible. Participation is voluntary.

Investigator's scoring sheet:

**During a typical month, how often do you eat these food items?**	Never	Seldom	1-2 times per month	3-4 times per month	2-3 times per week	>3 times per week
Herring for dinner?	0	0	6	12	24	48
Fatty fish like salmon, trout, sardine or mackerel for dinner?	0	0	4	8	16	32
Tuna, halibut, plaice or flounder for dinner?	0	0	2	4	8	16
Lean fish like redfish, catfish, pollack and cod for dinner?	0	0	1	2	4	8
Food made of fish paste, fish gratin and breaded fish for dinner?	0	0	1	2	4	8
Other seafood like crab, mussels, shrimps and lobster for dinner?	0	0	2	4	8	16
Fatty fish (herring, salmon, sardine and anchovy) as bread spread?	0	0	4	8	16	32
Lean fish (cod, tuna and food made of fish paste) as bread spread?	0	0	0.5	1	2	4
Other seafood (crab, shrimps and crayfish tails) as bread spread?	0	0	0.5	1	2	4
Cod liver oil or omega-3 in liquid or capsulated form?	0	0	3	6	12	24

Total intake of omega-3 fatty acids per month: _ _ _ , _ gram
