# Supplementary material for: Marine n-3 fatty acid consumption in a Norwegian renal transplant cohort: Comparison of a food frequency questionnaire with plasma phospholipid marine n-3 levels
Source: PLoS One. 2020 Dec 17;15(12):e0244089. doi: 10.1371/journal.pone.0244089 (PMC7746258; doi:10.1371/journal.pone.0244089)
Supplement: S3 Fig — The study subjects responded to the question “During a typical month, how often do you eat these food items?” using one out of six response alternatives for each food item categories. Based on EPA and DHA content in the meat of various fish and other seafoods presented in the US Department of Agriculture Food Composition Database and assuming a standard portion size for dinner and bread spread, every potential response was given a weight (shown inside boxes). Total intake of marine n-3 fatty acids per month was calculated as the sum of the weighted responses in grams, which for fatty fish intake comprised the four items shown. (PDF) [file pone.0244089.s003.pdf]

Food frequency questionnaire for the ORENTRA study

Randomization number								
Patient initials								
Date (day/month/year)					2	0		

In conjunction with your participation in the ORENTRA study, we ask you to answer the 10 questions below about your eating habits in regards to omega-3 fatty acid intake. Please choose only one answer per question. If you are uncertain about what you eat in a typical month, base your answers on your food intake in the past month. Please answer as honestly as possible. Participation is voluntary.

Investigator's scoring sheet:

**During a typical month, how often do you eat these food items?**	Never	Seldom	1-2 times per month	3-4 times per month	2-3 times per week	>3 times per week
Herring for dinner?	0	0	6	12	24	48
Fatty fish like salmon, trout, sardine or mackerel for dinner?	0	0	4	8	16	32
Fatty fish (herring, salmon, sardine and anchovy) as bread spread?	0	0	4	8	16	32

Total intake of omega-3 fatty acids per month: [ ][ ][ ] , [ ] gram
