# Supplementary figures and images for: Marine n-3 fatty acid consumption in a Norwegian renal transplant cohort: Comparison of a food frequency questionnaire with plasma phospholipid marine n-3 levels
Source: PLoS One. 2020 Dec 17;15(12):e0244089. doi: 10.1371/journal.pone.0244089 (PMC7746258; doi:10.1371/journal.pone.0244089)

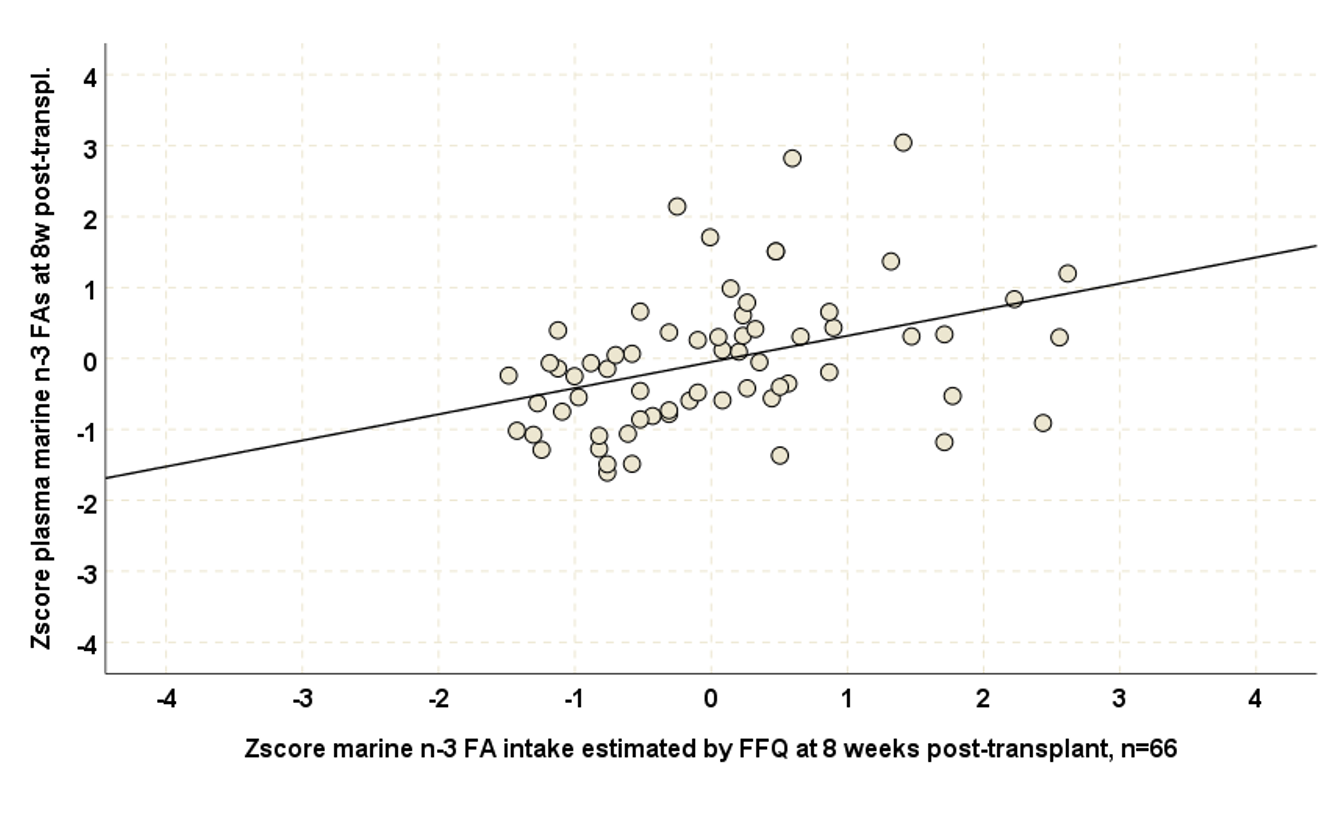

Supplement: S5 Fig — (TIF) [file pone.0244089.s005.tif]

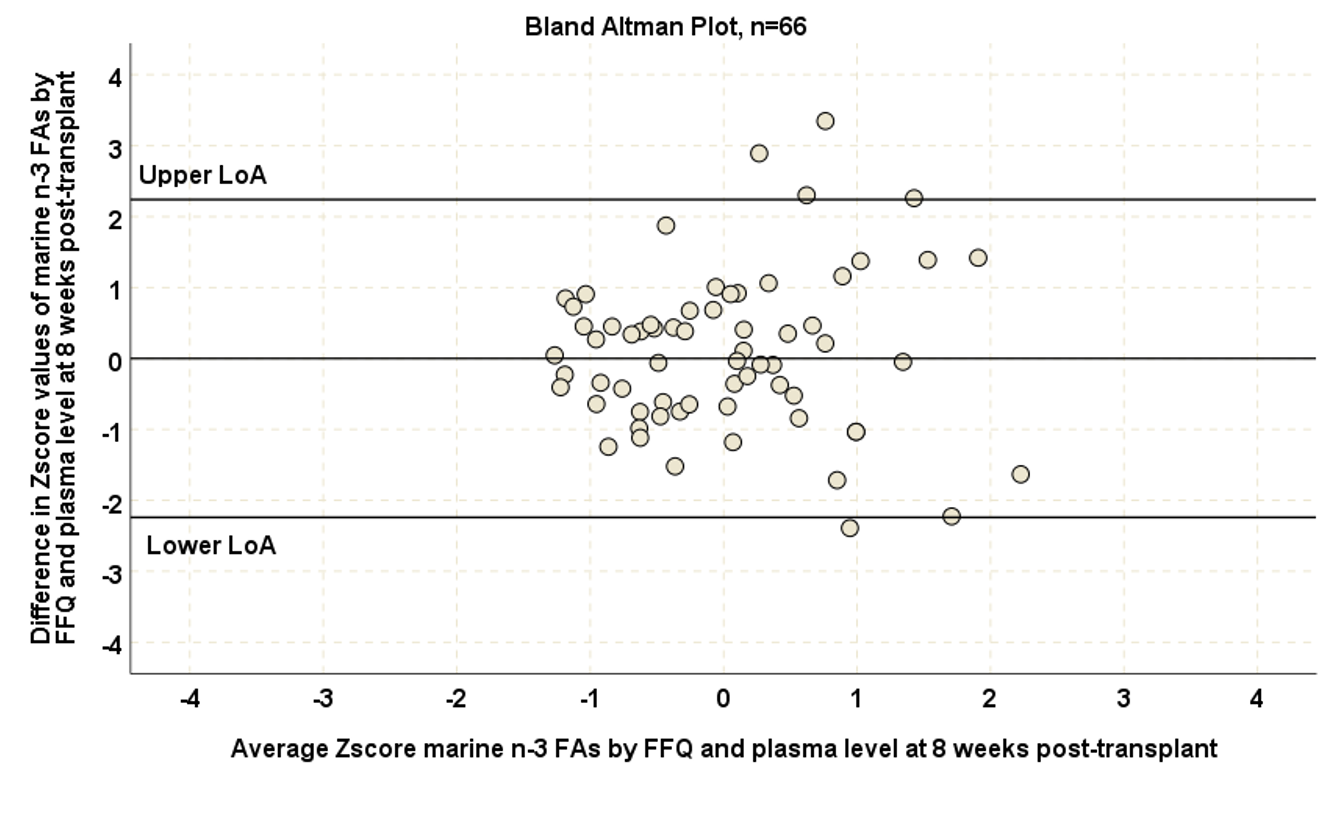

Supplement: S6 Fig — (TIF) [file pone.0244089.s006.tif]

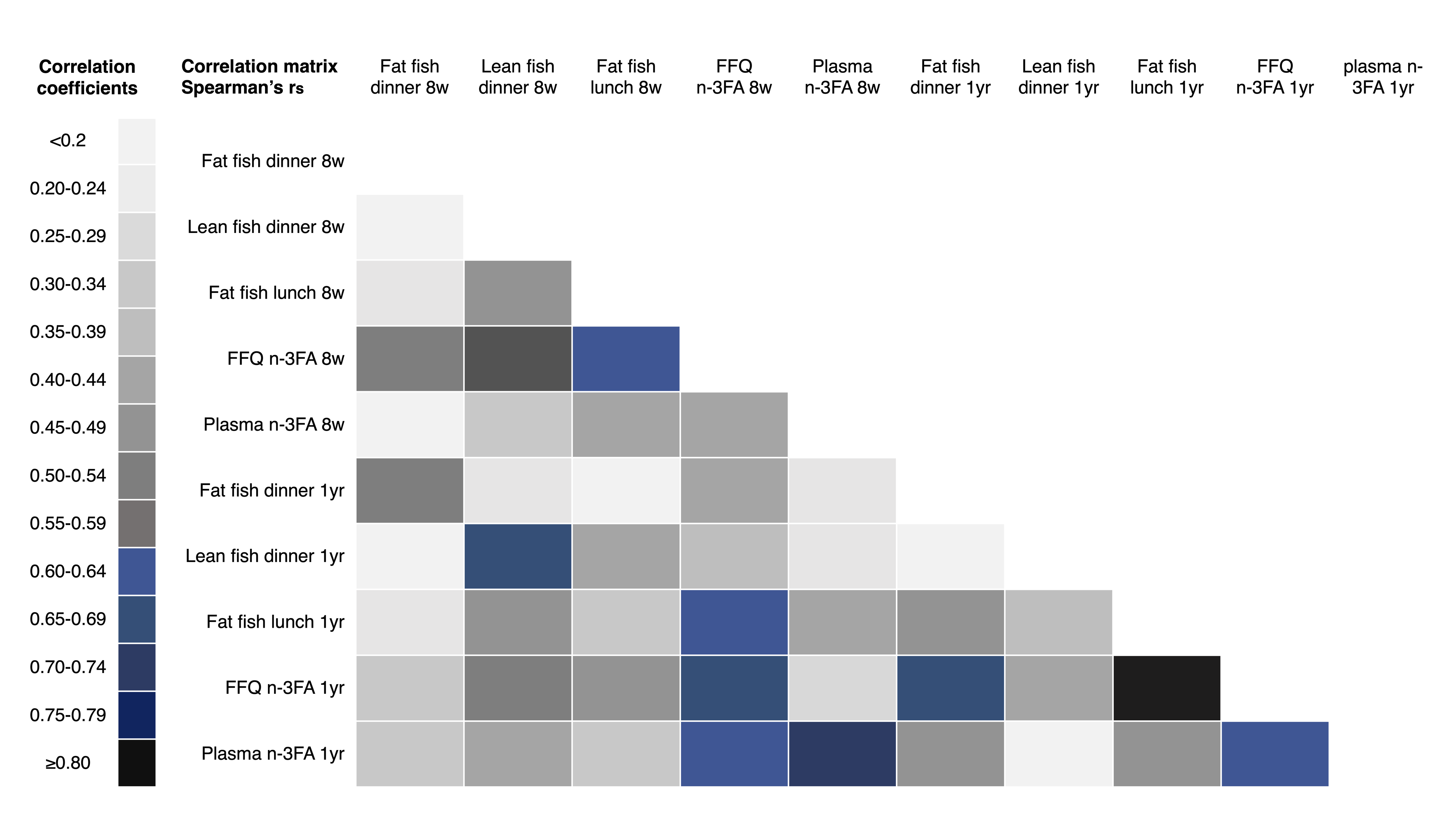

Supplement: S7 Fig — (TIF) [file pone.0244089.s007.tif]
