# Supplementary material for: Marine n-3 fatty acid consumption in a Norwegian renal transplant cohort: Comparison of a food frequency questionnaire with plasma phospholipid marine n-3 levels
Source: PLoS One. 2020 Dec 17;15(12):e0244089. doi: 10.1371/journal.pone.0244089 (PMC7746258; doi:10.1371/journal.pone.0244089)
Supplement: S1 File — Includes information regarding “Patient screening and recruitment in the ORENTRA trial”, “Fatty acid analysis”, “Sample Size and Power Calculation” and “Development of the study Food Frequency Questionnaire”. (DOCX) [file pone.0244089.s008.docx]

**Patient screening and recruitment in the ORENTRA trial**

The study was performed at Oslo University Hospital Rikshospitalet and patients were enrolled in the trial between 15^th^ of June 2013 and 15^th^ of June 2014. From 298 patients who received a renal transplant at this center in this period, 176 patients were eligible for inclusion (patients aged 18 years or more with a functional graft) and 132 patients were enrolled in the trial. Exclusion criteria were participation in another clinical trial, foreign citizenship, a kidney donor > 75 years old or allergy to fish or other seafoods. This study was an investigator initiated, single center, randomized, double blind, controlled trial, where validation of a food frequency questionnaire (FFQ) developed specifically for this trial was one of the secondary endpoints. At the baseline time-point associations between the FFQ based marine n-3FA consumption estimates and the reference biomarker plasma phospholipid marine n-3FA level were studied in the whole study cohort. At end of study only data from patients belonging to the study control group were used in the statistical analyses. Allocation concealment was ensured by the randomization method and study participants, care givers and investigators were kept blinded until the final monitoring visit at 25^th^ May 2017. There were no code breaks before blinding was stopped. Moreover, we used hard capsules of similar shape and color in both study groups, in order to avoid patients discovering whether they had received the study drug or the control oil. Data were entered into an electronic case report form at the study visits. At the final visit the electronic case report form and the study data file were locked, randomization codes were made available and statistical analyses could be undertaken.

**Fatty acid analysis**

Blood samples were drawn in a fasting state, centrifuged, frozen and stored at ‑80º C at the Renal Physiology Laboratory Biobank at Oslo University Hospital, Rikshospitalet, Norway.

Aliquots of plasma were sent to The Lipid Research Center, Aalborg University Hospital, Denmark, for fatty acid (FA) analysis, which consisted of four steps: Extraction of total lipids from plasma samples, phospholipid fraction isolation, phospholipid transmethylation and gas chromatography as described under:

1) Extraction of total lipids from plasma samples. Extraction of total lipids was performed by a modified Folch method [1]. Five hundred uL plasma was extracted with 5 mL of chloroform-methanol (2:1) containing 50 ug/mL butylated hydroxytoluene as antioxidant. After adding 0.75 mL of 0.9% sodium chloride, the tubes were mixed and centrifuged at 3220 g at 10º C for 10 minutes. The upper aqueous phase was discharged and the protein disk reextracted with 5 mL of chloroform-methanol (2:1) containing 50 ug/mL butylated hydroxytoluene and 1 mL 0.9% sodium chloride. The organic phases were combined and dried under nitrogen for 45 minutes at 40º C and dissolved in 1 mL of chloroform.

2) Phospholipid fraction isolation. The phospholipid fraction was isolated essentially as described by Burdge [2]. The lipids dissolved in 1 mL chloroform were transferred to a Bond Elut NH2 column (Agilent Technologies, US) preconditioned with 4 mL of hexane followed by washing with 4 mL of chloroform. The phospholipid fraction was eluted with 2 mL chloroform-methanol (3:2) followed by 2 mL of methanol, after which the phospholipid fraction was dried under nitrogen for 1 hour at 40º C.

3) Phospholipid transmethylation. Transmethylation of phospholipid FAs was performed after dissolving in 500 uL warm heptane (50º C), mixing briefly and then adding 25 uL of 2M potassium hydroxide in methanol and heating for 2 minutes at 50º C. After mixing, the tubes were centrifuged at 3220 g for 10 minutes at 10º C and the upper phase transferred to gas chromatographic injection tubes.

4) Gas chromatography. The FAs were quantitated using a Varian 3900 gas chromatograph with a CP-8400 autosampler, a flame ionization detector and a CP-Sil 88 60 m x 0.25 mm capillary column (Varian, Middleburg, The Netherlands). We used a split injection mode, constant flow rate, temperature programing from 90 - 210º C and helium as the carrier gas. Individual FAs were identified from their relative retention time, and quantitated as the weight percent of total fatty acids (wt%). This method allowed for quantification of FAs with 14-24 carbon atoms, including the two major marine n-3FAs eicosapentaenoic acid and docosahexaenoic acid.

**Sample Size and Power Calculation**

The study cohort consisted of patients who were enrolled in the ORENTRA trial (n=132). Power calculation for this trial was based on the primary endpoint renal function. The clinical steering committee discussed power calculations also for secondary endpoints, including for the study FFQ. Comparable studies used power estimations based on a correlation coefficient of at least 0.3 (95% CI and 20% drop-out rate was used for calculating sample size in these studies), necessitating a sample size of ≥ 84 patients. For r ≥ 0.4 we need ≥ 46 patients, for r ≥ 0.5 we need ≥ 29 patients and for r ≥ 0.6 we need ≥ 19 patients. Based on these reports, the sample size in the present study was adequate from a power perspective.

**Development of the study Food Frequency Questionnaire**

The study FFQ was developed in collaboration with dieticians at Oslo University Hospital Rikshospitalet. A pilot study was conducted before implementation of the study FFQ in the ORENTRA trial with 10 subject interviews. Standard portion sizes are based on information from Norwegian Food Safety Authority for a Norwegian population:

<https://www.matportalen.no/verktoy/the_norwegian_food_composition_table/weights_measures_and_portion_sizes_for_foods>

**References**

1. Folch J, Lees M, Sloane Stanley GH. A simple method for the isolation and purification of total lipides from animal tissues. The Journal of biological chemistry. 1957;226(1):497-509. Epub 1957/05/01. PubMed PMID: 13428781.

2. Burdge GC, Wright P, Jones AE, Wootton SA. A method for separation of phosphatidylcholine, triacylglycerol, non-esterified fatty acids and cholesterol esters from plasma by solid-phase extraction. The British journal of nutrition. 2000;84(5):781-7. Epub 2001/02/15. PubMed PMID: 11177194.
